# Race and gender differences in how sense of belonging influences decisions to major in STEM

**DOI:** 10.1186/s40594-018-0115-6

**Published:** 2018-04-10

**Authors:** Katherine Rainey, Melissa Dancy, Roslyn Mickelson, Elizabeth Stearns, Stephanie Moller

**Affiliations:** 1 Department of Physics, University of Colorado Boulder, UCB390, Boulder, CO 80309-0390 USA; 2 Department of Sociology, University of North Carolina at Charlotte, 9201 University City Blvd, Charlotte, NC 28223 USA

**Keywords:** Gender, Race, Belonging, Intersectional, Retention, Representation

## Abstract

**Background:**

Women and students of color are widely underrepresented in most STEM fields. In order to investigate this underrepresentation, we interviewed 201 college seniors, primarily women and people of color, who either majored in STEM or started but dropped a STEM major. Here we discuss one section of the longer interview that focused on students’ sense of belonging, which has been found to be related to retention. In our analysis, we examine the intersections of race and gender with students’ sense of belonging, a topic largely absent from the current literature.

**Results:**

We found that white men were most likely to report a sense of belonging whereas women of color were the least likely. Further, we found that representation within one’s STEM sub-discipline, namely biology versus the physical sciences, impacts sense of belonging for women. Four key factors were found to contribute to sense of belonging for all students interviewed: interpersonal relationships, perceived competence, personal interest, and science identity.

**Conclusions:**

Our findings indicate that students who remain in STEM majors report a greater sense of belonging than those who leave STEM. Additionally, we found that students from underrepresented groups are less likely to feel they belong. These findings highlight structural and cultural features of universities, as well as STEM curricula and pedagogy, that continue to privilege white males.

## Introduction

Women and people of color have been largely underrepresented in, and historically excluded from, most STEM fields, though trends are showing improvement (Kessel and Nelson, 2011; National Science Foundation and National Center for Science and Engineering Statistics, 2017). Reasons suggested for both race and gender underrepresentation span a range of factors including cultural norms, organizational structures, differential access to appropriate secondary school preparation, discrimination and harassment, and characteristics of individuals themselves. This literature is extensive. For the interested reader we suggest one of the National Academies’ summary reports on women and people of color in STEM (National Research Council, 2006, 2011, 2013). In this manuscript, we focus on one aspect of student experiences that have been connected to retention: student sense of belonging in their STEM field. We consider students’ self-reports of their sense of belonging in STEM in relation to their gender, race, and the intersections of those identities.

Our goal is to gain greater understanding of why there is persistent underrepresentation of women and students of color in STEM majors. To this end, we conducted in-depth interviews with 201 North Carolina college students from diverse gender and racial backgrounds. Based on information about their college majors provided to us in a screening survey that prospective interviewees completed, we selected individuals who were majoring in a STEM discipline or initially majored in a STEM discipline before switching to a non-STEM major. We label the former group as “majors” and the latter as “leavers.” Here we present a descriptive study of the experiences students indicated in their interviews as contributing either to their majoring in STEM or leaving STEM for another major. Specifically, we address the research questions:

1. To what extent do students of different genders and races report they feel they belong in their STEM field and what reasons do they give for belonging and not belonging?
2. How does sense of belonging in STEM compare for students who persist in STEM majors and those who leave?

This study is unique in several ways. First, we consider race and gender as well as their intersections, in contrast to most studies which consider only one dimension or the other. Secondly, our data come from a self-selected sample of college students with a wide range of academic and socio-economic backgrounds who attended 1 of the 16 public universities in North Carolina. North Carolina is a large state whose racial demographics mirror those of the USA. Many studies report data from predominately white institutions, only one institution, or from more selective institutions. The 16 campuses include small liberal arts colleges, STEM-focused institutions, and flagship research universities. They include historically Black colleges and universities and predominantly white institutions. Third, because representation of women and people of color is not consistent across STEM fields, we consider differences across different STEM fields instead of looking at only a single field or collapsing all majors into a single STEM category.

### Prior research

Sense of belonging refers to “students’ sense of being accepted, valued, included, and encouraged by others (teachers and peers) in the academic classroom setting and of feeling oneself to be an important part of the life and activity of the class” (Goodenow 1993, p. 80). Previous work indicates that sense of belonging in STEM has a significant impact on educational success and persistence, especially for women and students of color. Work related to sense of belonging in general and sense of belonging in STEM specifically is summarized below.

As Strayhorn summarized in his extensive review of research (Strayhorn 2012), sense of belonging is associated with academic achievement, retention, and persistence in college and these impacts are frequently more pronounced for students from marginalized groups. Recent studies have continued to confirm these findings (Smith et al. 2013; Thoman et al. 2014; Walton and Cohen 2011). Within STEM, both women and students of color have consistently reported less sense of belonging than men and white students (Good et al. 2012; Johnson 2012; Smith et al. 2013).

Socially stigmatized groups are more susceptible to belonging uncertainty (Walton and Cohen 2007), which arises when people feel unsure of their ability to “fit in” (Smith et al. 2013). Such feelings may cause those students, especially women, to experience competing belonging from non-STEM fields, pulling them out of STEM (Thoman et al. 2014). Fear of confirming negative stereotypes of a group one belongs to (gender, race, etc.) can undermine performance and contribute to a lack of sense of belonging as well. This can give rise to feelings that people like them do not belong there. Additionally, external cues, such as low representation of one’s group, can influence sense of belonging, particularly for women in male-dominated fields, such as most STEM disciplines (Murphy et al. 2007).

#### Factors contributing to sense of belonging

Peer interactions and interpersonal relationships have significant impacts on sense of belonging and are often seen as the most critical factor for overall sense of belonging (Johnson 2012). For example, engagement in peer discussions outside of the classroom was shown to increase the likelihood of women persisting in STEM (Espinosa 2011). Women of color, in particular, are highly influenced by the presence of peer relationships (Espinosa 2011; Weidman, 1989) and peer support overall (Espinosa 2011; Meiners et al. 2004; Shain 2002; Valenzuela 2006). Other factors have been shown to enhance students’ sense of belonging, and many of these have disproportionate effects on underrepresented students. For example, multiple academic and environmental interventions have been shown to improve women’s and minorities’ sense of belonging (Ramsey et al. 2013; Smith et al. 2013; Walton and Cohen 2011).

#### Gaps in the literature

Gaps in the STEM sense of belonging literature include the relative absence of research that addresses the intersection of gender with race. Research has shown that women struggle to maintain a positive sense of belonging in STEM class environments and that this lower sense of belonging in STEM is often associated with their loss of interest in the major (Good et al. 2012; Smith et al. 2013; Thoman et al. 2014).

Unfortunately, many studies regarding gender differences do not investigate racial identity, as many studies are done at predominately white institutions and people of color are notoriously underrepresented across STEM disciplines. Thus, often studies produce data about “women” that is only true for white women, leaving the experiences of women of color in STEM classrooms largely unexamined.

However, there are a few studies that look at the experiences of women of color regarding belonging (i.e., Johnson 2012; Mickelson et al. 2015), but this body of literature remains underdeveloped. Other studies about belonging in STEM fields that discuss racial identity often omit a gender analysis, additionally excluding women of color from the literature. There exist other intersectional works that investigate women of color’s experiences (e.g., Carlone and Johnson 2007; Crenshaw 1991), but we had difficulty finding literature that focused on women of color’s sense of belonging. In this manuscript, we attempt to fill this literature gap by explicitly discussing intersections of race and gender in our analyses.

#### Intersectionality

A major aspect of this study is an intersectional approach to data analysis. Intersectionality refers to the idea that aspects of one’s identity (e.g., race, gender, class, sexual orientation) are not unitary and mutually exclusive, but instead interact to construct one’s identity (Crenshaw 1991; Collins 2015). For example, considering race and gender as single axes of identity without analysis of the intersections of those groups can lead to the erasure of some identities, such as for women of color (Bowleg 2008; Crenshaw 1991). In this study, we look at two axes of identity—race and gender—and the intersections of these identities: white men, white women, men of color, and women of color. Though there are other axes that could be considered, these are the only axes of identity we recorded for our study. We acknowledge this limitation but believe that this intersectional approach will avoid the erasure of some students’ complex identities, such as those of women of color.

## Methods

This article reports findings from interviews collected as part of a larger mixed method study, the Roots of STEM Success Project (https://clas-pages.uncc.edu/rootsofstem/). Other analyses using the same dataset may be found in Mickelson et al. (2015) and Moller et al. (2015). The Roots of STEM Success Project was designed to study experiences in STEM of students who are traditionally not represented in STEM fields. The Roots project includes a large quantitative dataset with administrative data from middle school through college graduation of students who graduated from North Carolina high schools in 2004 and matriculated to one of the 16 campuses of the University of North Carolina (UNC) system. Many of these data are related to STEM success. In addition to the quantitative data, in 2013 the Roots Project conducted more than 300 interviews with college seniors who were asked to reflect upon their family, community, secondary school, and college experiences related to their decisions in choosing their college majors. In this paper, we focus on a subset of 201 of those interviews that were conducted with students who were either majoring in STEM fields or had declared a STEM major and left it for a non-STEM field.

We identified prospective interviewees by distributing an email recruitment survey to seniors at all 16 UNC campuses in January 2013. On these surveys, students could designate up to three major fields of study. Based on student responses, we categorized 201 respondents as STEM majors or STEM leavers (those who began in STEM but later elected to switch to a non-STEM major). To define a STEM major, we use the National Science Foundation Advance Program categorization (http://www.nsf.gov/crssprgm/advance/index.jsp) where majors such as engineering, physical sciences, earth, atmospheric or ocean sciences, mathematical and computer sciences, and biological and agricultural sciences are considered to be within the STEM category. We considered a student as a major if her or his current major, at the time of the interview, fell within these STEM fields. Given our interest in gender underrepresentation, we excluded social sciences from the definition of STEM fields. Additionally, in our analysis for this article, we look at differences across fields based on representation of women in the field.

We restricted our sample to students who attended public school (K-12) in North Carolina and who were younger than 30 years of age. These selection criteria were designed to align the interview sample with the quantitative data in the larger project. Students were asked to identify their racial/ethnic group on the survey. Once the potential interviewees were identified, we reached out to them via email to set up an interview (either by Skype, phone, or in person). We attempted to match interviewers with interviewees according to gender and race, although this was not always possible. The interviews lasted between 30 min and 1 h and were recorded for transcription purposes. Students were paid US$25 for participation in the interview.

### Interviewed sample

**Fig. 1:**
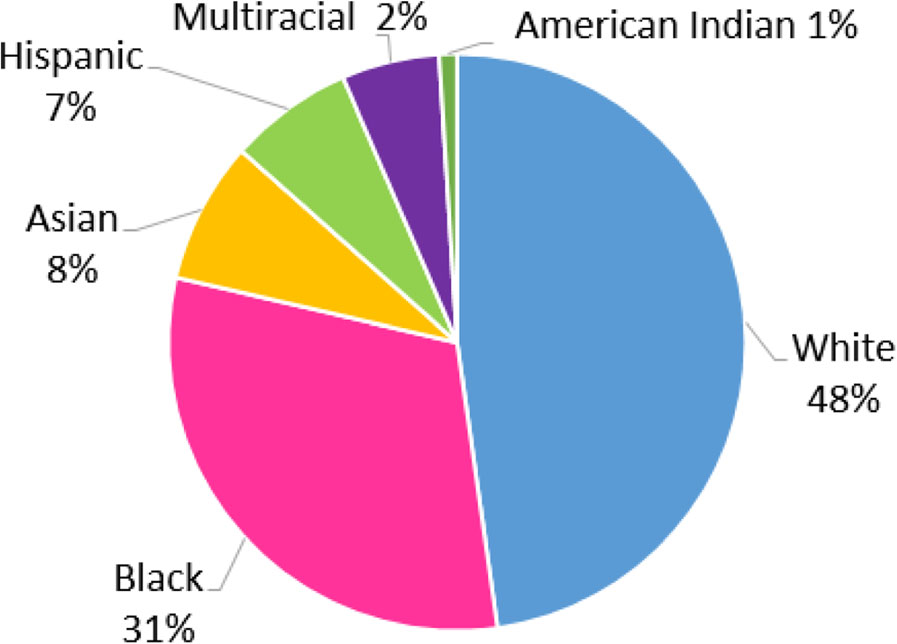
Demographics of interview students

In this article, we focus on interviews with 201 college seniors from the sampling frame of self-selected qualified volunteers. Because we oversampled students underrepresented in STEM, our purposive sample is not representative of STEM students in either race or gender. Of all students interviewed, 66% identified as women and 34% identified as men. Additionally, 48% of the students identified as white and 52% identified as students of color. The distribution of race among students interviewed is shown in Fig. 1. Race is based upon respondents’ self-identification. Given that we are interested in intersectionality and how it can help us better understand STEM outcomes, we disaggregate the sample by race, gender, and status (major vs. leaver).

### Interview protocol

The interview protocol was developed specifically for the Roots of STEM Success Project. Questions were designed to elicit a recounting of the participants’ history with STEM and factors that influenced their decision to major in STEM. We asked about family and peer influences, childhood informal educational experiences, secondary school and college experiences in and out of the classroom, beliefs about the self, attitudes toward STEM, and the students’ reasons for pursuing or not pursuing a STEM major.

### Interview analysis

We followed conventional procedures for analyzing the qualitative data captured in our interviews. Using a partial grounded theory approach to qualitative data analysis (Strauss and Corbin 1997), two researchers independently read the responses and coded under broad categories, some of which were determined a priori and others that emerged from the data. The researchers then compared their independently created codes. Through discussion, the codes were reorganized, collapsed, and expanded in a reiterative process until a coding scheme for the set of interviews was developed. Using the final coding scheme, all interviews were coded by two researchers and compared; discrepancies were aligned through discussion. Analysis and interpretation was done primarily by white women, with social and physical science backgrounds.

The presented findings focus on responses that were coded broadly as a student feels he/she “belongs in STEM” or “does not belong in STEM.” We applied these codes to any part of an interview in which a student made reference to belonging. Typically, sections coded as one of these two codes appeared as an answer to one or more of the three interview questions asked consecutively in the interview protocol:

1. Do you feel like you belong/belonged in {your STEM major}?
2. Did you ever feel out of place?
3. Has this feeling changed over time, and if so, what led to these changes?

Students reported either feeling they belonged or didn’t belong; others reported mixed feelings of belonging.

### Mixed methods analysis

In this paper, we present graphical representations of student’s reports of their sense of belonging based on our analyses of the interviews. We compare two specific groups with dramatically different responses or one particular group that stands out from responses of the rest of the respondents. Using a test of proportions, we calculated *Z*-scores for various comparisons as follows:

$$ Z=\frac{{\widehat{p}}_1-{\widehat{p}}_2}{\sqrt{\widehat{p}\left(1-\widehat{p}\right)\left(\frac{1}{n_1}+\frac{1}{n_2}\right)}}, $$

where ${\widehat{p}}_i$ is the proportion of people giving the analyzed response in relation to the total number of people in that group; subscripts 1 and 2 label the groups being compared. The $\widehat{p}$ without subscripts is the proportion of people giving the response in relation to the total number of people being compared and *n* represents the sample size. In every case where we report significance, we have used this test of proportions and calculated significance as a *z*-score. This *z*-score conveys the probability that respondents’ responses to interview questions are statistically independent of their various demographic characteristics. We acknowledge the limitations to the use of quantitative analysis of qualitative data and the debatable use of statistical tests of qualitative data (Maxwell 2010).

## Results

### The intersection of race and gender in students’ sense of belonging

First, we report student responses for sense of belonging in their STEM major by gender, race, and representation of demographic group in their STEM major. Students frequently gave multiple reasons for belonging or not belonging. Therefore, students’ overall belonging status was coded as follows: (1) belongs in STEM (positive belonging status, only reported feelings of belonging), or (2) does not belong in STEM (negative belonging status, only reported feelings of not belonging), or (3) mixed (reported both feelings of belonging and not belonging). This scheme permitted us to code all students uniquely into one of the three categories. All statistical results were determined using a test of proportions described above.

Not all students who reported their belonging status gave explanations for their sense of belonging while some gave multiple reasons. Because of this pattern, there is a discrepancy between the total number reported for belonging statuses and number of belonging explanations.

#### Effects of gender on belonging: women report less belonging

**Fig. 2:**
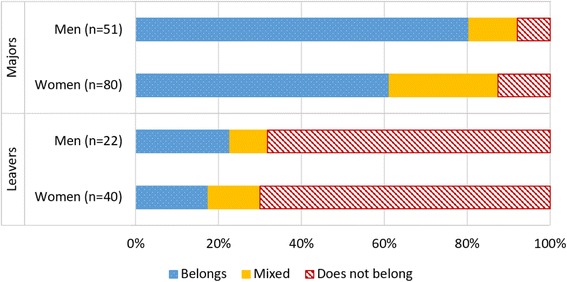
Belonging in STEM by gender and major status

We began our analyses by examining differences in belonging based on gender of participants. Results for both majors and leavers are reported in Fig. 2.

We have analyzable responses from 51 men and 80 women who were majors. We found that female majors were significantly less likely than male majors to report they felt they belonged in STEM (*Z* = 2.41, *p* ≤ 0.01), as can be seen in Fig. 2. We have responses from 22 men and 40 women who were leavers. We found similar results between male and female leavers. Not surprisingly, leavers were more likely to report they did not feel they belonged in the STEM field they left.

#### Effects of race on belonging: students of color report less belonging

**Fig. 3:**
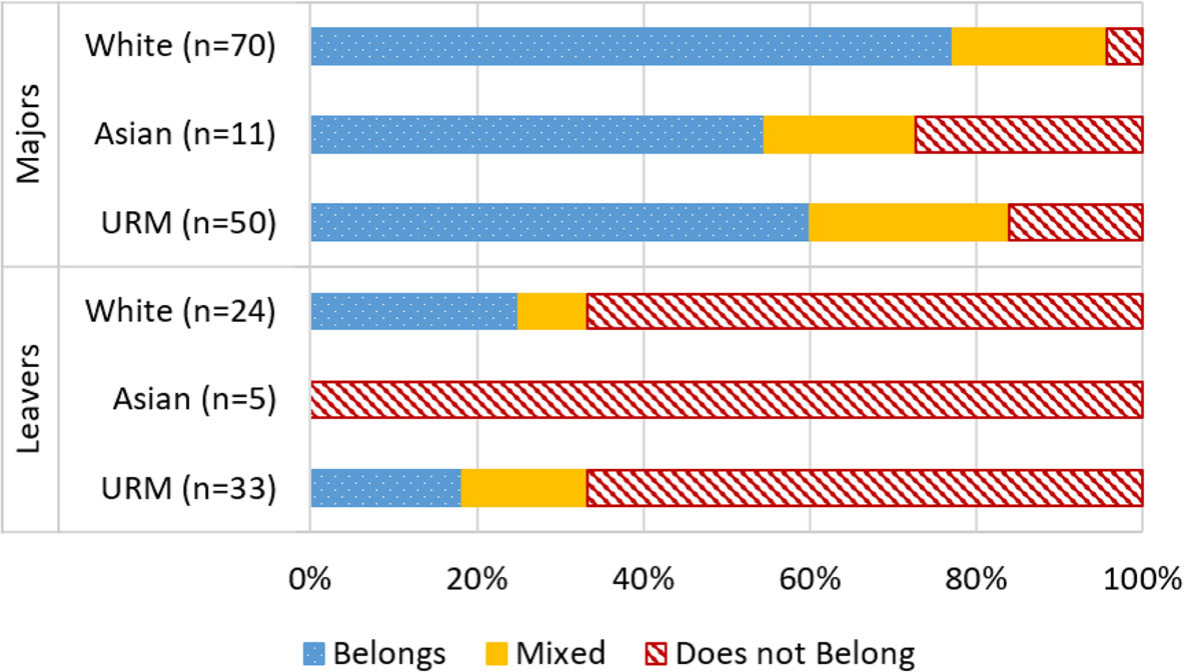
Belonging in STEM by race and major status

Next, we analyzed responses by the race of participants. Results for both majors and leavers are shown in Fig. 3. As mentioned above, there was a need to consolidate racial categories due to small numbers when dividing among many different groups. Here we report three racial groups: white students, Asian students, and underrepresented minorities (URMs). We report Asian students separately because while they are an ethnic minority, like the URMs, they are generally represented in STEM in proportion to their share of the population, as are white students (National Science Foundation and National Center for Science and Engineering Statistics 2017).

Among majors, we have responses from 70 white students, 11 Asian students, and 50 URMs. As shown in Fig. 3, we found that students of color who major in STEM are significantly less likely to report a sense of belonging than white majors (*Z* = 2.23, *p* ≤ 0.01). We have responses from 24 white students, 5 Asian students, and 35 URMs who were leavers. A large fraction of leavers from all demographic groups reported they did not have a sense of belonging in their STEM major.

#### Race and gender intersections

**Fig. 4:**
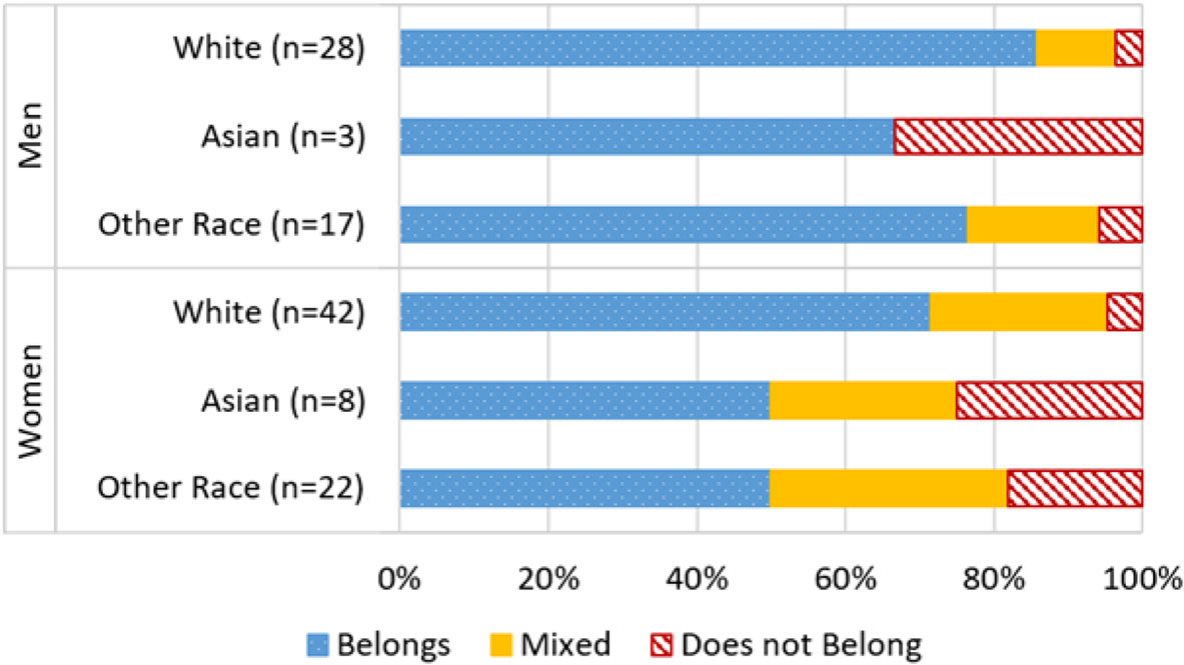
Majors’ belonging in STEM by gender and race

We next look at the relationships of belonging, race, and gender among majors, as presented in Fig. 4. Due to the smaller number of leavers and large frequency of a negative sense of belonging among those respondents, we focus only on majors in this section. Not surprisingly, with women reporting a lower sense of belonging than men, and students of color reporting a lower sense of belonging than white students, we find that women of color reported were the least likely to report a sense of belonging when compared to all other students (*Z* = 3.62, *p* ≤ 0.01), while white men were the most likely to report a sense of belonging when compared to all other students (*Z* = 2.37, *p* ≤ 0.01).

These results reflect the importance of simultaneously considering the intersections of gender and race in any analysis such as this study. While most majors feel they belong, when we consider both gender and race together, we see that most of the STEM majors who report perceptions of *not* belonging are men and women of color. Notably, they persisted in their STEM majors despite not feeling as if they belonged there.

#### Asian students and belonging

While the size of our racial subsamples are small, we note that the patterns for Asian students match those of URMs’ more closely than those of white students. This result suggests that although Asian students may be represented in STEM, their experiences may not align with those of the other ethnic group that is well-represented, white students. For the remainder of the paper, we collapse racial groups into only two categories: white students and students of color. We combine Asian students with other students of color because their responses (shown in Figs. 3 and 4) match closer to those of URM students than white students, and the number of Asian students in our self-selected sample is quite low.

#### Sense of belonging and representation of one’s demographic in the major

**Fig. 5:**
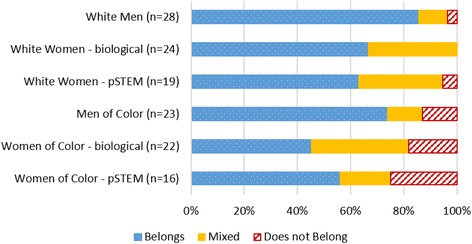
Belonging in STEM field by race and gender status

We examined sense of belonging within STEM fields by gender and race. We distinguish between biological science majors (biological) and the physical sciences (pSTEM). We do so because the representation of women varies between fields of study in STEM. In pSTEM, women are highly underrepresented, but in biological sciences, representation of women is close to or above parity at the undergraduate level. We present data for men without disaggregating STEM majors, as men are generally highly represented in all STEM fields. Figure 5 presents perceived belonging by women’s major. We find that women of color in pSTEM, where they are highly underrepresented, report belonging significantly less frequently than white men (*Z* = 2.17, *p* ≤ 0.05).

Due to small *n*, comparisons between groups should be made with caution. However, we note that there is a general tendency for feelings of belonging to follow patterns of representation. As a student’s demographic group becomes less represented, the less likely a person is to report a sense of belonging. We also note that lower sense of belonging was most commonly reported by people of color, suggesting that race significantly impacts belonging, perhaps even more than gender.

### Explanations for sense of belonging

We continue the analysis by looking at common factors cited for belonging and lack of belonging. We look at factors cited by majors for their reported sense of belonging (*n* = 70) and for a lack of a sense of belonging (*n* = 55). We also present factors cited by leavers for their low sense of belonging (*n* = 37). We omit explanation for leavers who felt they belonged in STEM because very few leavers reported a positive sense of belonging in STEM majors. Due to low numbers for majors and leavers, we present results based on race and gender, but not the intersections of those groups. It is noteworthy that not all interviewed students gave explanations for their belonging status while some gave multiple reasons.

Four broad themes emerged during the coding of answers regarding belonging, which we labeled as interpersonal relationships, science identity, personal interest, and competence. Students either had or lacked the aspects encompassed by the codes. For example, students can attribute their positive sense of belonging to having interpersonal relationships or attribute their negative sense of belonging to a lack of interpersonal relationships. It is worth noting that some students’ responses were labeled with more than one theme. Table 1 presents all belonging explanation codes.

**Table 1:** Summary of coding scheme used for belonging explanations. Each code is defined for students who had or lacked the reason described

Code	Reason for belonging	Reason for not belonging
Interpersonal relationships	Feels socially connected with peers and/or faculty members. May share common interests with peers.	Lacks a social connection with peers. Feels socially different, does not fit in.
Science identity	Science is a part of their identity as a person.	Lacks a personal connection to the major or material.
Personal interest	Expresses personal interest in course subject or major.	Explicit lack of interest. May find the material boring or unrelated to their reason for choosing their major.
Competence	Feels like they understand major-related material or receives good grades in major-related courses.	Feels like they do not understand major-related material well or receives poor grades in major-related courses.

#### Interpersonal relationships and belonging

In his theory of undergraduate socialization, Weidman defines interpersonal interaction as one of the three processes of socialization. This includes relationships with peers or faculty as well as the frequency of interactions and intensity of those relationships (Weidman 1989). In this study, the code interpersonal relationships (IRs) encompasses any personal relationships that students have with other members of their associated STEM department. Having IRs means that a participant feels socially connected or similar to those around them in their STEM major. For example, one student coded as having IRs explained,

*I can really relate to the other biology majors. Most of my friends are biology majors. I feel like it’s where I belong. – White female biology major*

Another interviewee related IRs and belonging to knowing people in the major, as can be seen in his response to the question about feeling out of place:

*At first I was just starting to get used to everything because I didn’t know everyone but now I just fall right in*.

*– White male information technology major*

These students were both coded as having IRs in STEM that contributed to their positive sense of belonging in their major.

On the other hand, a lack of IRs indicates a lack of social connection or similarity to those around them. Responses for a lack of IRs included differing hobbies from peers and social isolation, among other things. For example, one student coded as not belonging due to “lacks interpersonal relationships” said that, though he enjoyed the course work associated with his STEM degree, the social environment made him uncomfortable:

*I enjoyed the classes. I just did not enjoy the atmosphere. When I looked around, I saw all these people, all these people that I didn’t fit in with, and I didn’t feel comfortable there. – White male exercise science leaver*

An Asian female leaver described how sometimes the lack of belonging was related to her demographic status:

*[I felt out of place] especially because I was like 1 of 2 girls at the time that was a Physics major. Even that other girl that was a Physics major with me, I think she changed to a math major. – Asian female physics leaver*

#### Science identity and belonging

In contrast to connections developed through interpersonal relationships, science identity is more focused on the individual student. Science identity as defined in this study is related to one’s personal connection to their field, meaning science is closely connected to their sense of self. Our definition overlaps in part with Carlone and Johnson’s definition of research scientist identity, which relates to excitement for uncovering the natural world and scientific knowledge (Carlone and Johnson 2007; Espinosa 2011). In short, science identity in our study encompasses one’s feeling of being a “science person.” Participants who expressed belonging based on having a positive science identity describe their major as an integral part of their life and who they are. When asked about whether he felt he belonged in his field, one student responded

*Absolutely. I feel like this is exactly where I belong, and this is the type of work that I want to do, and I feel like everybody in my major kind of sees eye to eye on the same issues in technology that I do.*

*– White male engineering technology major*

Here, this student expresses that engineering technology is “exactly” where he belongs and what he wants to do, conveying that it is an integral part of his life. Students with a science identity, like the major quoted below, expressed feelings of their major being a part of who they are:

*[I feel like I belong in engineering because] it’s what I do and it’s kind of becoming who I am. So it’s kind of like… taking your major and becoming what it is. – Black female systems engineering major*

This major expresses a personal connection to her major in stating she is “becoming what [her major] is.” Similarly, a biology major equated her life with her major:

*As much as I complain about my major I couldn’t see myself learning anything else. My life is biology. – Black female biology major*

These responses exemplify “science identity” because the interviewees are connecting their major to their sense of self.

In contrast, many who lacked a science identity expressed feeling like there was no connection between the major and who they are as a person. When asked if he felt he belonged in his previous STEM major, one leaver answered “no” and explained:

*I didn’t feel like I was the type of person. Again, I’m not a nerdy guy. Not all scientific people are nerds, obviously, but I’m just a person who questions things just to understand, and that’s why I think I’m a lot better as a journalist because [what] you need to [do is] ask questions, and they weren’t people who answered questions well. – White male physics leaver*

Another leaver, in direct response to a question about belonging in her previous major, commented that she felt out of place due to her lack of passion:

*I definitely kind of felt a little weird because everyone that was around me was so much more excited about what we were doing than I was. And I kind of felt like that was a problem because if it was something that I really loved then I should be just as excited. – White female biology leaver*

#### Personal interest and belonging

While science identity is closely tied to students’ sense of self, personal interest relates to one’s interest in major-related material or the major in general. Typical responses coded in this category were “I enjoy it” and “the major fits my interest.” This differs from science identity because it lacks a connection to passion and sense of self. Responses categorized as personal interest focus on interest in the field, independent of how they view themselves as a person. Someone who was coded as having a science identity made a personal connection between themselves and the field, whereas someone coded as having personal interest expressed interest in a way that was not connected to who they are as a person. For example, as a direct response to the question about belonging, one participant said she belongs when she is interested in the material and does not feel she belongs when she is not interested:

*When I was in the classes I care about I feel like I belonged but when I’m classes that’s like, we’re talking about plants, vertebrate zoology, and stuff I feel like I don’t belong there. – Black female biology major*

Here, she doesn’t express having or lacking passion, but instead a lack of interest in a particular subfield that diminishes her sense of belonging. Both majors and leavers expressed a lack of personal interest as a factor contributing to their lack of belonging. A lack of personal interest expressed by majors was often attributed to an emphasis in the degree program that differed from what they were interested in. For example, a biology major cited a lack of personal interest because the degree program focuses on anatomy and genetics while her personal biology interests lean more towards marine biology. One student expressed a similar perspective when asked if she felt she belonged:

*To be quite honest, not really… I have learned a lot of math but realizing that I am more applied math, the math department at [my school] on the whole is not an applied math department. It is much more theory-based.*

*– White female math major*

Here, this major encountered a discrepancy between the content she is interested in and the content she encountered; she lacked personal interest in the content emphasized in her department, which contributed to her perception of not belonging in the major.

#### Competence and belonging

In addition to interest in the subject matter, feelings of belonging or lack of belonging were also influenced by students’ perceived competence in the subject matter. Competence in this study refers to people’s perception of their performance and understanding. This definition aligns with Carlone and Johnson, who defined competence as one’s perceived grasp of scientific concepts and material (Carlone and Johnson 2007; Espinosa 2011). Competence is captured by grades, conceptual understanding, and ability to communicate understanding to others. Participants frequently cited competence as a reason why they belonged. For example, when asked if she felt she belonged in biology one major replied,

*I do now. (laughs) Because I know that I can have a good understanding of everything I’ve been learning and it’s like I know that because I can teach others. I can help others understand. – Black female biology major*

Numerous students cited their struggle to understand concepts as a reason why they did not feel they belonged. When asked if she ever felt out of place, one woman said she sometimes felt out of place or uncomfortable and gave the explanation:

*[My classmates had] a lot of practical knowledge and ... I didn’t have all that knowledge and I was trying to learn it... I think that was a struggle for me.*

*– Hispanic female computer engineering leaver*

This leaver felt that her understanding of the material did not measure up to that of her peers; her feeling out of place was due to a lack of competence. Another student’s explanation for his lack of sense of belonging echoes the previous student’s explanation:

*I feel out of place because I think some of [the other majors] know more than I do and I wonder how because we have taken the same classes.*

*– Black male information technology major*

Another student described mixed feelings of belonging that varied depending upon his self-perceived competence at the moment:

*Sometimes I do feel out of place, for example, with that group project… I didn’t really know that much, but with another group project… I felt like I belonged because I had good ideas and contributed to the group and people listened to me… It kind of varies. – Black male information technology major*

It should be noted that students’ responses about poor grades may not align with common expectations of what a poor grade is. For example, several female STEM leavers we interviewed considered receiving any grade less than an A in a class as a bad grade. Thus, a high-achieving student may report low competence despite getting high grades in their STEM courses and would be coded as lacking competence. Our coding of competence is based on students’ perception of their own understanding and performance; we did not have access to information regarding students’ actual grades or course performance.

#### Frequency of themes

**Fig. 6:**
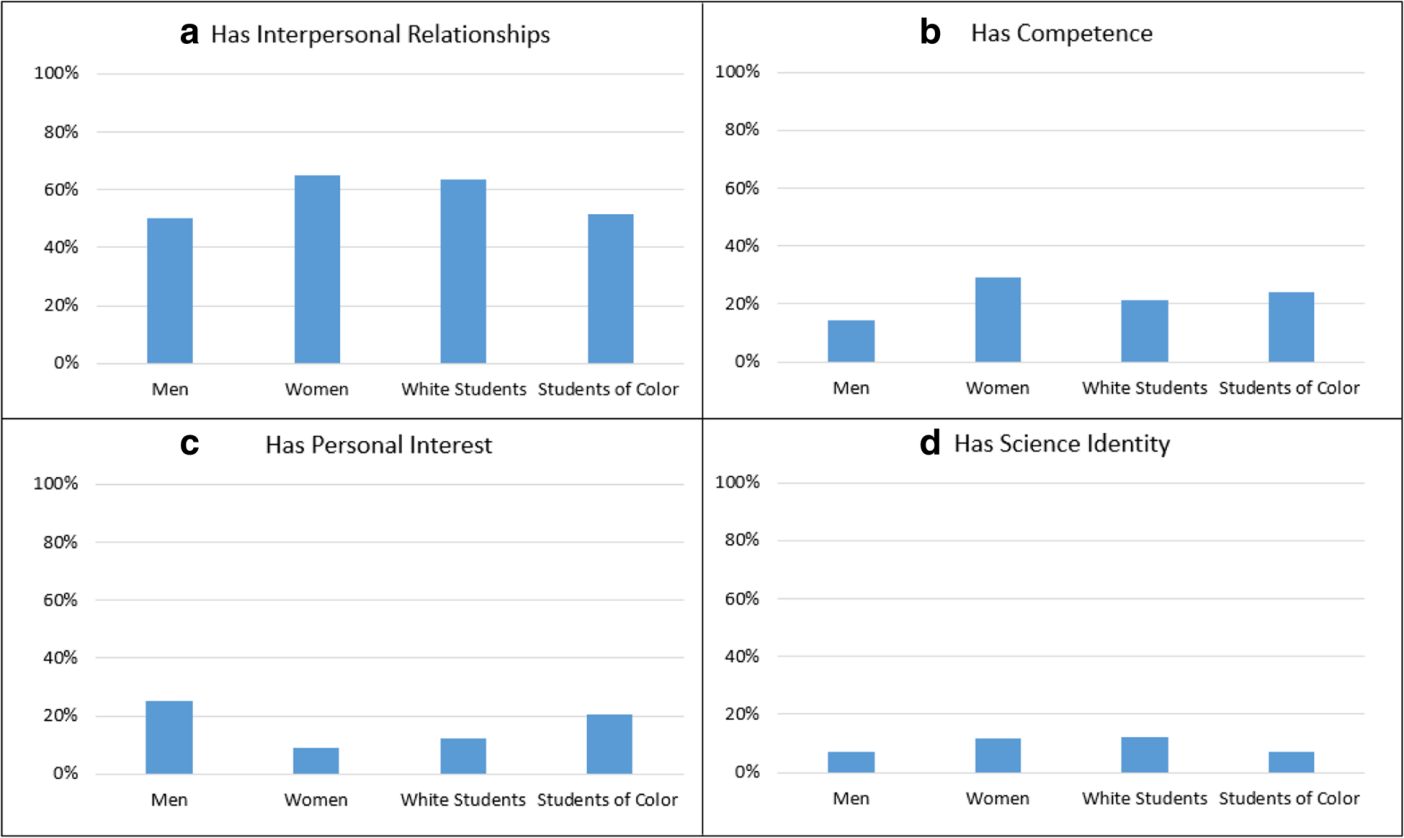
Reasons majors cite for belonging in STEM by race and gender

In order to gauge the frequency with which these themes appeared in the interviews, appearance of each code was counted for majors and leavers. Figure 6 shows common responses for positive sense of belonging among STEM majors. Students could offer several reasons for belonging. Interpersonal relationships were the largest factor cited for each demographic group, aligning with the literature (Strayhorn 2012). Competence was the second most commonly cited factor for majors’ positive sense of belonging and was cited at similar frequencies for all demographic groups. Personal interest was the next most frequently cited by majors. Science identity was cited by only a small percent of interviewees. There were no significant differences between responses of men and women or white students and students of color.

**Fig. 7:**
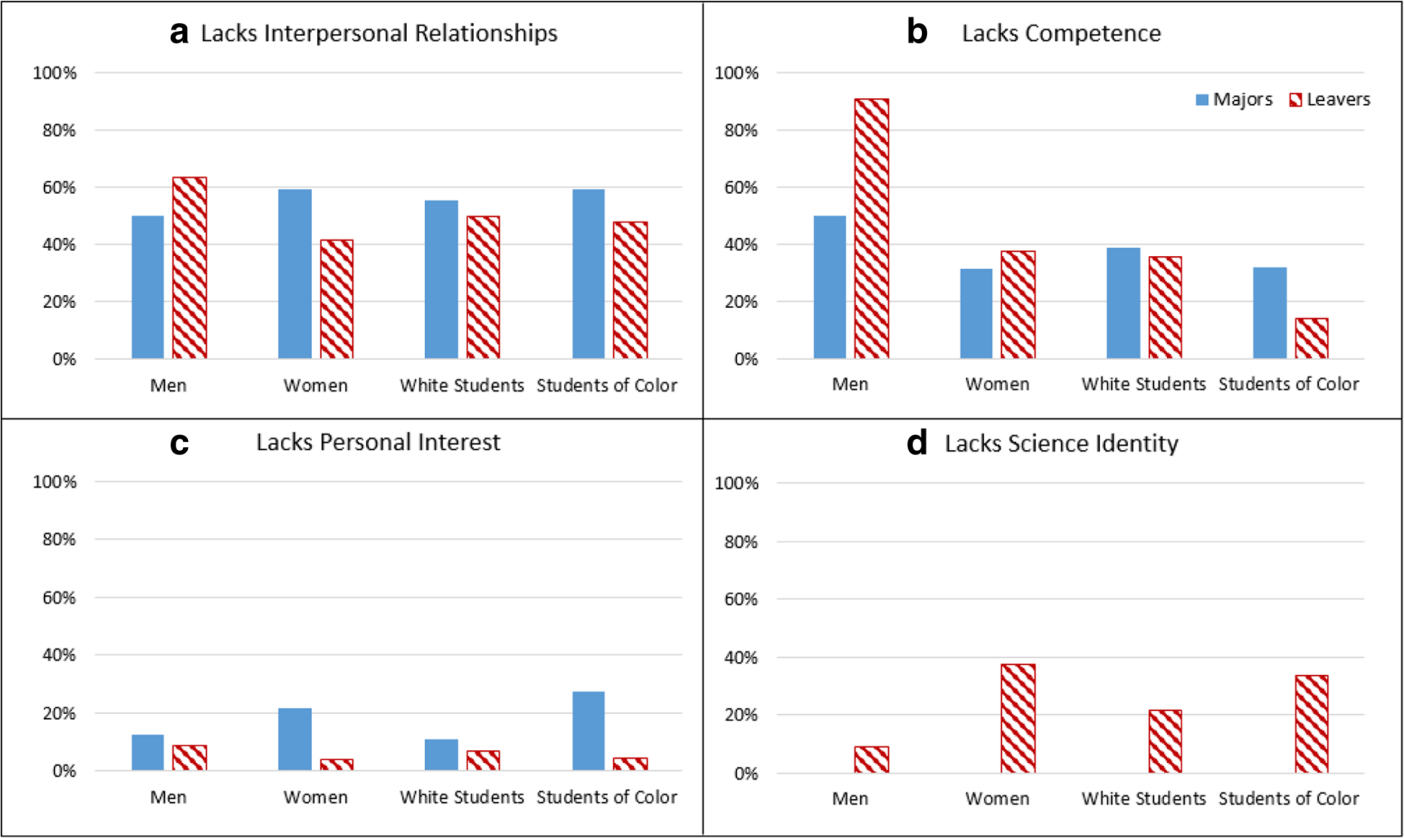
Reasons majors and leavers cite for not belonging in STEM by race and gender

Figure 7 shows common responses among majors and leavers who reported a lack of a sense of belonging in STEM. The most frequently cited factor contributing to a lack of belonging among majors was the absence of interpersonal relationships. This was true for all demographic groups. Lack of competence was the second most frequently cited explanation for all majors. No majors cited a lack of science identity as an explanation for their negative belonging status. Absence of interpersonal relationships was the most commonly reported factor contributing to leavers’ negative belonging status. Leavers rarely cited lack of personal interest for their sense of not belonging in their STEM major.

## Discussion

Prior research and this study suggest that for many students, particularly women and underrepresented minorities, a sense of belonging in one’s STEM major can be a necessary although not sufficient condition for students’ success. In this study, we examined self-reported sense of belonging and found that these feelings are associated with having personal relationships with college peers, students’ confidence in their abilities to complete the requirements of the major, their interest in their major field, and whether or not the student has a science identity. We found that sense of belonging was reported more among men, white students, and majors. We also found that STEM students’ sense of belonging is correlated with the number of members of the students’ gender who also are in their major. The visible presence of learners “like me” renders the student’s presence in this STEM environment as normative rather than an aberration—essentially conveying to the learner that “I belong” here.

When students addressed the issues of whether or not they belong in their STEM major, they most commonly cited one of four reasons:

1. The presence or absence of meaningful interpersonal relationships with others in the major.
2. The presence or absence of a sense of competence in the major.
3. The presence or absence of a personal interest in the major subject.
4. The presence or absence of a personal sense of science identity.

### Majors

Interviewees who reported personal relationships with others in the major were less likely to leave it. The influence of a personal relationship on staying or leaving the major holds for women, white students, and students of color. Across race and gender categories, perceived competence was the second most commonly cited reason given for feelings of belonging and/or lack thereof. Majors are more likely to attribute their science identity as a reason for their sense of belonging (as opposed to a lack of it being a reason for not belonging) and remaining in their major. Levels of personal interest in the STEM discipline contribute to a small degree to major’s sense of belonging, but its magnitude is not markedly different than levels among leavers, with the exception of women and students of color, whose personal interests contribute more to their reasons for belonging than interest contributes for men and white students.

### Leavers

The main reasons leavers give for their low levels of belonging are a lack interpersonal relationships and weak sense of competence. Interviewees cited lack of confidence in their capacities to complete the major at a certain level as a motivation for leaving. However, unlike majors, leavers also report a lack of science identity as a significant reason for not belonging. And this lack of science identity is more prevalent for leavers who are women and students of color than for other leavers.

### Implications

#### Intersectionality matters

Research that examines only race or only gender differences in outcomes may mask the ways intersections of race and gender shape experiences of students. Our interviews indicate that both race and gender moderate the experiences that impact sense of belonging for science students. Importantly, we see large differences between women of color and both white women and men of color. Women of color reported the feeling a sense of belonging less frequently than any demographic group. Only a bit more than half of the women of color majors reported consistent feelings of belonging. These were women who were nearing graduation with a STEM major and yet frequently did not feel they belonged in the field in which they were about to receive a degree. The extent to which this group struggles with belonging can be overlooked when race and gender are not considered together. It is notable that so many women of color persisted in the major despite lacking sense of belonging. Though it is beyond the scope of this work, there exists literature (e.g., Ong et al. 2017) that investigates women of color’s persistence and resilience in STEM. This is an area that could benefit from further investigation.

Additionally, we see that the lack of belonging reported by men is primarily experienced by men of color. Combining the experiences of all men obscures this finding. Intersectional analysis is absent from the majority of the STEM education literature, which suggests that findings reported in the literature on women’s sense of belonging are most likely reporting the belonging status of white women and reports on people of color are most likely reporting the experiences of men of color. The unique experiences of women of color are obscured by this failure to consider both race and gender.

#### Demographic isolation is associated with lower sense of belonging

Analyses based on levels of gender or ethnic representativeness in a STEM field in relationship to students’ sense of belonging indicate that being demographically similar to others in the field positively impacts belonging. In fields where parity has been reached (i.e., white women in undergraduate biology programs), even students from demographic groups still underrepresented in other STEM fields report high levels of belonging. This raises the possibility that the problem is not that women or people of color inherently feel they do not belong in STEM but, rather, they are responding to the unbalanced representation of certain demographic groups in some fields of STEM. Such a response to demographic imbalance implies that even if all other individual, familial, or academic preparatory factors are aligned for a student’s STEM success, until STEM fields become more demographically diverse, those in the majority group (generally white males) will remain privileged by the culture and organization of the discipline in ways that sustain their sense of belonging while undermining the sense of belonging of students from underrepresented groups. This interpretation suggests the underlying problem is systemic (i.e., based in the cultures and organization of STEM rather than individuals’ characteristics or preferences) and will require a systemic solution crafted to change institutional cultures and organizational structures.

#### Science identity contributes to sense of belonging

The presence of a science identity differentiates those who stay and those who leave a STEM major. We find that the absence of a science identity is greater among underrepresented groups. No major indicated lack of science identity as a reason for not belonging. In contrast, approximately one quarter of leavers did so. Our science identity findings align with work by others who demonstrate the connection between science identity, race/gender, and persistence (Carlone and Johnson 2007; Hazari et al. 2013).

Our data is correlational, so we cannot determine the extent to which a lack of science identity motivates dropping out of STEM and the extent to which those who drop out then feel a lack of identity. However, among the leavers, women were more likely to report a lack of science identity than men, and students of color were more likely to report a lack of science identity than white students. No white male (major or leaver) reported a lack of science identity; all reports describing a lack of science identity were from students in marginalized groups. This relationship indicates that science identity is strongly connected to both race and gender and, given that students of color and women leave STEM at higher rates, it is likely that science identity plays a role in persistence.

#### Interest in science plays a role in sense of belonging

Leavers rarely cited their lack of personal interest in STEM to explain why they left their major. However, majors were more likely to mention it, especially students of color and women—even though interest in science contributed only a small part to interviewee’s sense of belonging. This is an important finding to note because it challenges the idea that women and people of color are inherently less interested in science and implies that structural and disciplinary cultural factors, rather than individual preferences, should be the focus of future examinations of representation in STEM.

#### Interpersonal relationships are essential

The relationships students form with faculty as well as with peers are important in relation to persistence. The presence or lack of interpersonal relationships was the most common reason cited for belonging or lack of belonging. This implies that for the department interested in increasing persistence rates among students from all demographic backgrounds, taking inventory of the extent to which members of the department—faculty and students—form positive connections and providing structures that encourage strong interpersonal relationships are likely to be productive strategies.

## Conclusions

Women and people of color continue to be largely underrepresented in most STEM fields. Multiple explanations have been offered for this underrepresentation. Here we focus on students’ sense of belonging in their STEM major, one of many aspects of student experiences that previously have been shown to be related to retention. In particular, we considered students’ self-reports of their sense of belonging in STEM in relation to their gender, race, and the intersections of those identities.

Using a series of interviews with undergraduates from diverse ethnic backgrounds, we sought to gain greater understanding of why there is persistent underrepresentation of women and students of color in STEM majors. Our finding that leavers across all demographic groups report a lower sense of belonging indicates an association between feelings of belonging and persistence in STEM. We additionally find students from underrepresented groups are less likely to feel they belong. By considering the intersections of race and gender, we illuminate this particularly striking association among women of color, who are more likely than any other demographic group to feel they do not belong in STEM, a sentiment we found even among women of color who persist in the field. The final set of findings addresses the major reasons student cite for belonging in their major: presence or absence of interpersonal relationships, personal interest in the field, a sense of competence, and whether or not the individual has developed a science identity.

Our study is unique in several ways. First, as mentioned previously, we consider race and gender and their intersections in contrast to most studies which consider only one dimension or the other. Secondly, our data come from a self-selected sample of college students with a wide range of racial and academic backgrounds. Many studies report data from only one institution or from more selective institutions. Our data better represents the general college population in the state of North Carolina, which is itself highly representative of the nation itself. Third, we consider differences across different STEM fields instead of looking at only a single field or collapsing all majors into a single STEM category. This illuminates the impact of representation in a given field on feelings of belonging and the need to consider STEM fields independently.

While these strengths distinguish our research, we are aware of several limitations of the study. First and foremost, our sample is self-selected individuals from a purposive sampling frame we developed: college seniors in North Carolina who both graduated from a North Carolina high school and attended one of the 16 UNC campuses. Second, because of the limitations of our interview data, we could only investigate two axes of identity: race and gender. Thus, our sample restricts any generalizability of our findings beyond the sample. However, our carefully crafted sample offers many important insights into the larger dilemma that informed the study. Our findings generate avenues for future empirical work that can tests the insights from this study.

White men who pursue STEM benefit from the privileges of being in a field in which there are many others similar to them and where they frequently fit stereotypes about who pursues STEM. Even when students stated they belonged in STEM they sometimes expressed feeling others did not feel they belonged due to not fitting a stereotype. For example, a white female biology major stated “I think sometimes you have to work a little harder to be taken seriously as a blonde female.” Additionally, having commonalities with others privileges white males in forming interpersonal relationships, which are shown to be important for persistence. Students from underrepresented groups sometimes described difficulties engaging in group work where the rest of the group was entirely white men.

Our work suggests a need to identify and better understand the ways in which the organization, norms, and cultural climates of STEM disciplines work to support those in the privileged group while often discouraging those from marginalized groups. In the biggest of pictures, taken together, our findings point toward cultural and structural factors that impact sense of belonging for STEM students, which in turn, can impact persistence.
